# The Impacts of Sugar-Sweetened Beverages (SSB) on Cardiovascular Health

**DOI:** 10.7759/cureus.26908

**Published:** 2022-07-16

**Authors:** Dylan Pietrantoni, Harvey N Mayrovitz

**Affiliations:** 1 Medicine, Nova Southeastern University Dr. Kiran C. Patel College of Osteopathic Medicine, Clearwater, USA; 2 Medical Education, Nova Southeastern University Dr. Kiran C. Patel College of Allopathic Medicine, Davie, USA

**Keywords:** vascular, heart, coronary, cardiovascular, sugar-sweetened beverage

## Abstract

Cardiovascular disease (CVD) has been a prominent global health challenge in the last decade, and many risk factors and outcomes of CVD have been studied in that timeframe. Recent research has explored the association between sugar-sweetened beverage (SSB) consumption and CVD; however, there is a lack of updated reviews regarding SSB consumption impacts on CVD outcomes and the possible mechanisms affecting the disease state. In turn, this review aims to summarize the relevant published research from the last decade regarding linkages between SSB consumption and CVD outcomes and the potential underlying mechanisms, as well as to highlight opportunities for future exploration with respect to those outcomes and mechanisms. In this review, we searched PubMed, Embase, and Web of Science for peer-reviewed articles published from January 2012 to March 2022 regarding SSB consumption and its association with CVD.

The results of our search reveal strong evidence that the consumption of SSB is positively associated with increased risks of CVD and that the magnitude of that risk is increased in a dose-dependent manner. These increased risks range from elevated triglyceride levels to inclined risk of CVD-related mortality. Although the depth of the mechanisms responsible for these increased risks have been less explored thus far, there is some evidence supporting SSB implications in cardiovascular factors, including vascular function, coronary artery calcification, triglyceride levels, inflammatory processes, arterial stiffness, and genetic polymorphisms.

## Introduction and background

Sugar-sweetened beverages (SSB) comprise a vast array of liquid drinks, including fruit juices, carbonated sodas, vitamin water drinks, energy drinks with added sucrose, fruit juice extracts, and, most commonly, high-fructose corn syrup [1]. Most of these are common staples of daily fluid consumption in the United States of America (USA). SSB may offer a unique and dangerously easy mechanism of sugar ingestion. One 360 ml serving of soda, for example, contains about 36 grams of sugar [2]. Daily ingestion of that one serving could alone add five pounds of weight gain in one year if the diet does not accommodate for the additional sugar load [2]. While it is still unclear which components of SSB drive the overall negative impacts on health, most recent studies have shown that there is a correlation between SSB consumption and cardiovascular disease (CVD) [3-5].

CVD has been a prominent global health challenge in the last decade [6]. Research in that timeframe has shown that SSB consumption is positively associated with CVD occurrence worldwide and has been reported to be a significant contributor to cardiometabolic-related deaths in the USA in 2012 [3,5,7]. Other studies have examined factors affecting the relationship between SSB consumption and CVD, such as dose dependence, gender-specific occurrences, and consumption frequency [4,8,9]. Most of this research has focused on outcome-based metrics (e.g., mortality or myocardial infarction occurrence) across different populations [10,11]. Although much uncertainty remains as to the mechanisms responsible for the link between SSB consumption and CVD, some studies evaluating potential mechanisms, such as vascular function, coronary artery calcification, triglyceride involvement, inflammatory processes, changes in arterial wall compliance, and genetic variation, present compelling information for review and possibly highlight future research opportunities [8,12-19]. A gap exists in updated reviews of literature focused on CVD outcomes and possible means leading to the disease state. Thus, the remainder of this article is dedicated to reviewing studies regarding SSB consumption on the spectrum of the aforementioned CVD outcomes and potential underlying mechanisms.

## Review

The goals of this review were to summarize relevant published research regarding linkages between SSB consumption and CVD and to highlight opportunities for future research to clarify mechanisms behind the CVD outcomes associated with SSB consumption. For this purpose, use is made of risk comparisons of morbidity and mortality outcomes for persons consuming various amounts of SSBs as reported in the literature. These assessments often utilize relative risk parameters and hazard ratios to quantify the findings.

Search strategy

Three databases were searched for peer-reviewed articles written in English: PubMed, Embase, and Web of Science. The phrase “sweetened beverage*” was mandatory in the title and “sugar” was mandatory in the title or abstract of the searches. Additionally, one or more of the following words was mandatory in the title: “cardi*”, “coronar*”, “heart”, “vasc*”, “myo*”, “infarct*”, “ischem*”, “stroke”, “hypertension”. The purpose of the asterisks (*) was to include any titles with variations of those words. Studies published from January 2012 to March 2022 were included. After accounting for duplicate articles across the three databases, 71 initial articles remained for review. After exclusion of two animal studies, 19 review and systematic review articles, and 26 research articles with non-CVD focuses (i.e., effects of policy changes or taxation on a population’s SSB consumption, non-alcoholic fatty liver disease, obesity), 24 meta-analyses and primary articles remained for review. Additionally, six more relevant articles found within the initial 24 studies were included for a total of 30 articles assessed in this review.

The impacts of global SSB consumption on CVD occurrence and mortality

The link between SSB consumption and CVD occurrence and mortality has been studied from several different perspectives. One way was to compare the health outcomes of sugary beverage consumers versus non-consumers [20]. In this study, data was collected on the French population through dietary questionnaires and official medical records across a 10-year span (2009-2019) from 104,760 participants with no previous history of CVD. A high sugary beverage was defined as having at least 5% sugar. The top third sugary beverage consumers had significantly higher risks than non-consumers for first CVD events, including stroke, myocardial infarction, and transient ischemic attack (p=0.009). Other studies have shown similar results in populations outside of France [11,21]. An analysis of the Singapore Chinese Health Study showed a higher risk of acute myocardial infarction in participants (men and women aged 45-75) that consumed one or more servings of SSB daily versus non-consumers after adjusting for energy intake and BMI with an odds ratio of 1.93 (95% CI: 1.23, 3.00) [11]. Another study in Japan found a significantly greater risk for ischemic stroke between women who consumed SSB every day or almost every day versus women who never or rarely consumed SSB (p=0.02) [21]. One meta-analysis of seven studies, including both male and female participants across several countries, found a 9% increased risk of CVD development with each additional daily SSB serving [4]. Similarly, another meta-analysis of 11 studies and almost 17,000 CVD events found an 8% higher risk of CVD occurrence with each additional serving of SSB per day [22]. These findings further support a deleterious SSB-CVD linkage.

Specific research on cardiovascular mortalities associated with SSB consumption also supports the SSB-CVD linkage. For example, an analysis of over 100,000 men and women found a 31% higher risk of death from CVD in persons who consumed two or more SSB servings per day versus those who consumed less than one serving per month (p<0.0001) [23]. Another meta-analysis reported a 13% increase in cardiovascular-related death in higher SSB consumers versus non- or limited-SSB consumers [24]. Other research has shown dose-dependent relationships between CVD-related mortality and SSB consumption similar to those previously discussed on CVD events. Consuming just one 250 ml serving of SSB per day was associated with an increased relative risk (RR) of CVD-related death (RR 1.06; 95% CI: 1.00, 1.12; p<0.001) [3]. The same study also showed a stronger statistical significance of CVD-related death with SSB consumption greater than 500 ml per day (RR: 1.24; 95% CI: 1.16, 1.31; p<0.001). These studies, along with others from the past decade, provide evidence of an SSB association with the spectrum of diseases classified as CVD. We only found one study within the search criteria that reported opposing results. This study evaluated 338 daily consumers of sugar-containing soft drinks versus 1231 very low consumers (less than one soft drink per month) and did not show an association between the SSB consumers and vascular events, including strokes, myocardial infarctions, and vascular deaths [25].

Associations of SSB consumption with specific cardiovascular events

Because CVD encompasses a large span of illnesses affecting the heart and vasculature, it is of interest and potential utility to focus more specifically on these varied aspects individually.

SSB Impacts on Coronary Vascular Disease

Coronary-specific diseases have been investigated over the last ten years. The presence or history of myocardial infarction (MI) is one outcome measure by which studies gauge coronary artery disease (CAD). Some evidence suggests an increased risk of CAD for high versus low SSB consumers based on MI risk. One study found in a 22-year span of initially 18,000 participants that the top-quartile of SSB consumers had a 20% higher risk versus the bottom-quartile of developing CAD after accounting for lifestyle variables, including age, smoking, exercise, body weight changes, and diet considerations (RR 1.20; 95% CI: 1.09, 1.33; p<0.01) [8]. Two other meta-analyses calculated similar results with increased risks of CAD (RR 1.17; 95% CI: 1.07, 1.28) and MI (RR 1.19; 95% CI: 1.09, 1.31), respectively, in high- versus low-consuming SSB groups [26,27]. Another approach to investigate SSB-CAD associations examines dose-dependence. A study that included nearly 300,000 persons who were followed for eight years reported that consuming an additional 355 ml SSB per day was associated with a significantly increased hazard ratio (HR) for MI in men (HR 1.10; 95% CI: 1.02, 1.17) and in men and women combined (HR 1.08; 95% CI: 1.02, 1.14) [28]. Similarly, another study that included more than 40,000 subjects reported that the risk of coronary heart disease increased from 19% to 25% with each additional daily SSB serving (p<0.02) [8]. These studies are consistent with a dose-dependent relationship between the amount of SSB consumption and CAD.

More limited evidence points to a link between SSB consumption and coronary disease-related deaths. An analysis of the United States National Health and Nutrition Examination Survey (NHANES) across 15 years that included more than 30,000 individuals reported a significant difference in heart disease deaths between the highest and lowest quintiles of SSB consumers (HR 1.45; 95% CI: 1.06, 1.97) [29]. The data also indicated that heart disease deaths were dependent on the amount of SSB consumed. However, aspects of the data acquisition process in this study make it prudent to interpret these results with caution. The results included deaths from multiple International Statistical Classification of Diseases (ICD) codes pertaining to heart disease, and only a subset of these codes included direct diagnoses of CAD, and these were not separately considered in the analysis. The Global Burden of Disease Study 2017 considered data spanning 27 years and assessed mortality in China from ICD diagnoses more aligned with CAD [30]. However, these results assessed only differences in mortality in age stratifications of high consumers and did not provide direct, strong support for an SSB-CAD linkage. One further reference of coronary-specific cause of death in relation to SSB consumption lacked statistical significance [28]. Although reports concerning linkages of SSB consumption specifically to coronary artery disease were limited, the available evidence based on SSB consumption versus non-consumption and on dose-dependent risk increases support for the concept that SSBs play an increasingly negative role in coronary health.

SSB Impacts on Stroke

One aspect of peripheral vascular disease (PVD) regards the potential impact of SSB consumption on stroke. A study of 106,178 women in the California Teachers Study found a significantly higher hazard ratio for stroke in those who consumed one or more servings of SSBs daily versus those who rarely or never consumed SSB (HR 1.21; 95% CI: 1.04, 1.41) [31]. This association is consistent with results from a meta-analysis based on 3.5 million individuals [24]. They reported that SSB high-consumers were at significantly greater risk of stroke versus non- or low-consuming cohorts (RR 1.12; 95% CI: 1.03, 1.23). Another study based on an analysis of 70,000 men and women aged 45-83 years old found that risks of both cerebral infarction (RR 1.22; 95% CI: 1.04, 1.42) and total stroke (RR 1.19; 95% CI: 1.04, 1.36) were significantly elevated with groups consuming greater than 400 ml of SSB per day versus those who consumed less than 100 ml per day [32]. Because of the large number of persons included, the statistical power in these three studies provides persuasive evidence towards an association between SSB consumption and risk of stroke. Stroke risk has also been reported to depend on SSB dose [27]. Although most data are consistent with an association between SSB consumption and stroke, we found no results on separate SSB consumption with deaths due to stroke. In the three previously mentioned studies of increasing stroke incidence, deaths due to stroke were not parsed from stroke events [24,31,32]. Thus, the lack of information on relationships between SSBs and these deaths, apart from the disease occurrences, exposes a gap, potentially warranting future investigation.

SSB Impacts on Blood Pressure

The effects of SSBs on blood pressure have also been investigated. In one study of over 60,000 university students in Iran, individuals in the highest category of SSB intake were at 2.17 times greater risk of developing hypertension versus a low-consumption group (OR 2.17; 95% CI: 1.91, 2.47) [33]. Similarly, in a meta-analysis of approximately 240,000 individuals, the highest consumers of SSB with greater than 6.7 oz per day intake had a 12% greater risk of hypertension than non-SSB consuming participants (RR 1.12; 95% CI: 1.06, 1.17) [34]. These findings are consistent with an association between SSB consumption and hypertension development. Another meta-analysis gives insight to the quantitative impact of SSB on blood pressure in young people [35]. The results from over 90,000 participants 19 years old or less found a weighted mean difference (WMD) of 1.67 mmHg increase in systolic blood pressure (SBP) in participants with high consumption of SSB (WMD 1.67; 95% CI: 1.021, 2.321; p<0.001). Several studies reported that SSB consumption impacts blood pressure in a dose-dependent manner. One meta-analysis that included approximately 56,000 participants reported hypertension risk increased by 10% with each additional 250 ml per day of SSB consumed (RR 1.10; 95% CI: 1.06, 1.14; I2=58.4%) [36]. Another meta-analysis also found a dose-response feature with a reported increased risk of hypertension of 8.2% with each added daily SSB serving [34]. One limitation in assessing the SSB-hypertension link is the impreciseness of the term “hypertension.” One study alludes to the standards for hypertension in children and adolescents, who were the focus of their research [35]. However, none of the studies provide clear, numerical inclusion criteria. As meta-analyses, they used results of individual sub-analyses to determine the classifications of hypertension. This ambiguity may be an opportunity for future meta-analyses to clarify inclusion criteria more plainly and uniformly, further strengthening the statistical evidence. Even with this limitation, the presented studies still suggest a relationship and correlation between hypertension and SSB intake.

Markers and mechanisms of SSB-CVD linkages

While studies investigating SSB-CVD linkages are relatively numerous, few studies address the potential mechanisms involved. These include possible impacts of SSB on endothelial cell properties or vascular function, triglycerides, C-reactive protein (CRP), and other biomarkers. These aspects are considered in this section.

SSB Impacts on Vascular Function

Only one small study was found that addressed this issue directly by comparing the impacts of SSB versus water consumption on endothelial cell properties in healthy males [16]. This study utilized iontophoresis for the assessment of microvascular endothelium and vascular smooth muscle function. The process of iontophoresis includes delivering a charged pharmacological solution to the skin and examining the vascular response via laser speckle contrast imaging, which is a common non-invasive approach to measuring microvascular blood flow in clinical trials [37]. The two drugs used in the iontophoresis method of this study were acetylcholine and sodium nitroprusside, which are standardly used to assess the dilatory component of the microvasculature [16,37]. Further, macrovascular assessment included the measurement of brachial artery diameter according to the International Brachial Reactivity Task Force Guidelines, a standard consisting of high-resolution vascular ultrasonography [38]. Flow-mediated vasodilation and nitrate-mediated vasodilation are two measures of macrovascular function in this protocol.

Following a 10-hour fasting interval, 12 male subjects consumed 600 ml of water over a five-minute interval during one visit and 600 ml of a commercial SSB in another five-minute interval at a separate visit [16]. Fifteen minutes after beverage consumption, skin blood flow responses to iontophoresis of acetylcholine and sodium nitroprusside were evaluated. Immediately thereafter, flow-mediated vasodilation and nitrate-mediated vasodilation were measured. Compared to water consumption, they found that SSB consumption reduced the normal perfusion increase associated with acetylcholine iontophoresis (208.3 ± 24.3 versus 144.2 ± 15.7%, p<0.01) and also reduced the normal magnitude of flow-mediated dilation (0.019 ± 0.002 versus 0.014 ± 0.002%; p<0.01). No significant effect of nitroprusside or nitrate-mediated vasodilation was found. This study was important in opening the idea to further research on SSB impacts on endothelial cells and other aspects of vascular function beyond its acute hyperglycemic effects as outlined in its methods [16].

SSB Impacts on Triglycerides

Another possible SSB-CVD linkage is its involvement with triglycerides [8,12,17]. A study of Australian adolescents (14-17 years old) reported 7.0-8.4% increases in triglycerides when participants increased SSB consumption well above their baseline consumption levels [12]. A larger study of 42,883 male healthcare professionals showed a significant positive correlation between SSB intake and plasma triglycerides [8]. Further evidence of an SSB-triglyceride connection was reported for a hospitalized inpatient population [17]. The study included persons consuming beverages with high-fructose corn syrup (HFCS) concentrations of 0%, 10%, 17.5%, or 25%, respectively. Results showed a linear dose-response increase in triglyceride levels (p<0.0001). Taken together these reported findings provide support for an association between triglycerides and SSBs and possible ties to cardiovascular health overall.

SSB Impacts on C-Reactive Protein

Several studies have reported positive associations between SSB intake and C-reactive protein (CRP) [8,15,18]. Because CRP is an inflammatory biomarker with evidenced linkages to vascular dysfunction, this may be a relevant parameter for investigation [39]. A study of over 8000 female registered nurses reported that more frequent SSB drinking was positively correlated with CRP levels (p<0.002) [18]. Another study analyzed NHANES data of children three to 11 years old from 1999 to 2004 and reported a significant positive association between SSB consumption and CRP blood levels (p=0.003) [15]. An aforementioned analysis of data from health professionals reported a similar positive correlation between SSB intake and CRP levels (p<0.02) [8]. Such findings point toward a potential SSB-CRP linkage with possible connections to vascular effects requiring further study.

SSB Impacts on Coronary Artery Calcium

A positive association between SSB intake and the amount of coronary artery calcium (CAC) has also been reported [13]. CAC is usually detected by cardiac computed tomography, and the amount is quantified via a standardized protocol [40]. The significance of these scores relates to the potential risk of coronary artery occlusion and myocardial infarction [41]. Because of this possible linkage between SSB intake and elevated CAC scores, this measure may be of clinical importance. A study of more than 22,000 men and women of median age of 40 years reported significantly higher CAC scores in the highest SSB-consuming cohort (more than five SSB drinks/week) versus non-SSB drinkers (CAC ratio: 1.70; 95% CI 1.03, 2.81) [13]. In contrast to these findings, a study of 1991 men and women with a mean age of 55 years failed to show a significant association between SSB intake and CAC scores, even amongst the highest consumers of more than two drinks per day [42]. Various factors could have contributed to the different results of the two studies. One difference was the population of each study. The study supporting the association examined data from a population within hospital health centers in South Korea between 2011 and 2013 [13]. The other study assessed data from the National Heart, Lung, and Blood Institute Family Heart Study, which first examined patients in the early 1990s and recorded CAC scores between 2002 and 2003 [42]. Another factor contributing to the different findings may have been the sample sizes of each study; the study supporting a positive association between SSB and CAC scores included 22,210 participants [13]. In contrast, the analysis without a significant association included 1,991 participants [42]. An additional area of difference was the method by which the studies grouped the participants. One study grouped participants into five categories of SSB consumption: none, less than or equal to one, one to three, three to five, and five or more drinks per week) [13]. The other study used the following groups: almost never, one to three drinks per month, one per week, two to six per week, one per day, and two per day for stable estimates [42]. Further research on this issue may support one of these two results.

*SSB Impacts on Arterial Stiffness Determined via Pulse Wave Velocity Measurements* 

Arterial pulse wave velocity (PWV) depends on the stiffness of the arteries in which the pulses are traveling with a higher velocity indicating a greater stiffness [43]. Such measurements can be made non-invasively using one of several commercial devices, and evidence supports the measurement as a prediction for cardiovascular risk [44]. Thus, an impact of SSB consumption on this parameter may be of clinical interest.

One study used this method to investigate a possible association between fructose consumption, including SSB, and carotid-femoral PWV measurements in over 1000 participants [14]. The researchers analyzed data from the Brisighella Heart Study by categorizing qualifying participants into four groups: low fruit and low SSB intake (LFLB), high fruit and low SSB intake (HFLB), low fruit and high SSB intake (LFHB), high fruit and high SSB intake (HFHB). Low fruit intake was defined as less than two portions per day, and low SSB intake was defined as less than one drink per day. The analysis showed significant increases in PWV of the LFHB group compared to the LFLB (p<0.05) and HFLB (p<0.05) groups. There were also significant increases in PWV of the HFHB group compared to both the LFLB (p<0.05) and HFLB (p<0.05) groups. The significant differences between both high-consumption SSB groups versus both low-consumption SSB groups provides compelling grounds to further expand on the research of PWV and SSB intake.

Role of Genetic Variations in the SSB-CVD Link

Studies have shown an association between single nucleotide polymorphisms (SNPs) on chromosome 9p21 and CVD [45,46]. In an analysis of 1560 persons with myocardial infarctions versus 1751 healthy controls, a significant association between SSB intake of more than two servings/day and SNPs was detected in one of the variants of chromosome 9p21 (OR 1.44; 95% CI: 1.19, 1.74) [19].

This study elucidates the opportunity for expansion of research on the association between SSB intake and genetic involvement. Although the study included an appreciable sample size of 1560 participants with CVD events, the population was specific to Hispanics living in Costa Rica [19]. Replication of this study’s methods in other populations could further support or introduce contradiction to the association between SSB intake and SNPs in variants of chromosome 9p21. Further, this study focused on the variants of chromosome 9p21 with respect to SSB consumption based on prior associations with CVD [45,46]. This concept could be expanded to more research on the effects of SSB intake with identified genetic components with increased CVD risks. Continued investigation in this realm could help identify the most at-risk individuals and populations for CVD occurrence with SSB intake, providing the opportunity for health professional intervention and preventative measures.

## Conclusions

There is strong evidence that the consumption of SSB is positively associated with increased risks of CVD and that the magnitude of those risks are increased in a dose-dependent fashion with SSB intake. Specifically, support exists for increased risk of stroke, myocardial infarction, and CVD-related mortality associated with the consumption of SSB.

Research focuses on both CVD outcomes and the mechanisms responsible for the disease state in relation to SSB intake can be expanded to continue the depth of this knowledge. For example, within the vast spectrum of populations consuming SSBs that are affected by CVD, there may be certain ethnicities, races, communities, etc., with significantly worse CVD outcomes. Further investigation into the associations of SSB consumption with CVD outcomes in different populations and continued expansion of the methods in which these studies are performed can improve the current knowledge. Similarly, the current finite research on mechanisms underlying SSB involvement in CVD presents many avenues for future studies. Compounding research into vascular function, coronary artery calcification, triglyceride effects, inflammatory processes, arterial stiffness, and genetic polymorphisms in SSB-consuming participants could increase our understanding of the drinks’ implications in CVD. Examples may include expanded studies with PWV measurements of different SSB-consuming populations or dose-dependent effects of the sugary drinks on genetic variations with CVD associations.

This review brings attention to recently studied associations between SSB intake and CVD outcomes and possible mechanisms leading to those outcomes. It also illuminates opportunities for future research in the field. The information highlighted throughout this article may serve to bring awareness to the SSB-CVD linkages and aid in interventions to improve health outcomes.
